# *Lactobacillus rhamnosus* GR-1 Prevents *Escherichia coli*-Induced Apoptosis Through PINK1/Parkin-Mediated Mitophagy in Bovine Mastitis

**DOI:** 10.3389/fimmu.2021.715098

**Published:** 2021-09-14

**Authors:** Yanan Li, Yaohong Zhu, Bingxin Chu, Ning Liu, Shiyan Chen, Jiufeng Wang

**Affiliations:** Department of Veterinary Medicine, China Agricultural University, Beijing, China

**Keywords:** mastitis, *Escherichia coli*, mitophagy, NLRP3 inflammasome, ROS, *Lactobacillus rhamnosus*

## Abstract

*Escherichia coli* is one of the most important pathogens that cause clinical mastitis in dairy cattle worldwide and lead to severe economic losses. Antibiotics are often used to treat this inflammatory disease; however, antimicrobial resistance and environmental pollution cannot be ignored. Probiotic is the best alternative; however, its mechanisms of action to prevent mastitis remain unclear. Moreover, the role of probiotics in regulating mitophagy, a selective autophagy that maintains mitochondrial quality, needs to be explored. *E. coli* infection induced NOD-like receptor family member pyrin domain-containing protein 3 (NLRP3) inflammasome assembly, Caspase-1 activation, and apoptosis in MAC-T cells. Infection also resulted in mitochondrial damage and subsequent increase in reactive oxygen species (ROS) production. Moreover, inhibition of ROS release by scavenger N-acetyl-L-cysteine (NAC) abrogated the importance of ROS in NLRP3 assembly and apoptosis in MAC-T cells. Pretreatment with *Lactobacillus rhamnosus* GR-1 (LGR-1), a probiotic, alleviated *E. coli*-induced NLRP3 inflammasome activation and apoptosis *via* ROS inhibition. Besides, *E. coli* infection inhibited mitophagy while LGR-1 pretreatment augmented PINK1/Parkin–mediated mitophagy activation, which further blocked ROS generation. To explore the effect of LGR-1 *in vivo*, a mouse mastitis model was established. The results showed that LGR-1 pretreatment had preventive and protective effects on *E. coli* induced mastitis, and could reduce cytokines levels such as IL-1β and TNF-α. In accordance with the results *in vitro*, *E. coli* can inhibit mitophagy and activate NLRP3 inflammasome and apoptosis, while LGR-1 can weaken the effect of *E. coli*. Taken together, our data indicated that LGR-1 pretreatment induced PINK1/Parkin-mediated mitophagy that eliminated damaged mitochondria and reduced ROS production and NLRP3 inflammasome activation, which subsequently decreased *E. coli*-induced apoptosis. To conclude, our study suggests that therapeutic strategies aiming at the upregulation of mitophagy under *E. coli*-induced mastitis may preserve mitochondrial function and provide theoretical support for the application of probiotics in bovine mastitis.

## Introduction

Mastitis, mainly caused by a microbial infection, is the inflammation of the breast parenchyma associated with lactation (1). In dairy cattle, mastitis, which causes swelling and pain in the udder and systemic inflammatory injuries, seriously affects animal health and reduces milk yield and quality, leading to considerable economic losses worldwide (2). Clinical bovine mastitis caused by *Escherichia coli* (*E. coli*) leads to endotoxin shock and death in extreme cases (3, 4). Although many countries have improved farm management practices and reduced the incidence, mastitis caused by *E. coli* has proven difficult to solve (5). So far, antibiotics have been used to treat mastitis. However, this approach often leads to environmental pollution, bacterial resistance, and antibiotic residues, which affect human health, increase veterinary care costs, and result in premature slaughter (6, 7). Therefore, it is necessary to find a target and a new drug or any other alternative to prevent and treat mastitis in dairy cattle.

Probiotics have gained special attention as an alternative to antibiotics. They are defined as “live microorganisms which when administered in adequate amounts confer a health benefit on the host” (8). Microbes recognized as intestinal probiotics include Lactobacillus, Bifidobacterium, Streptococcus, and few *E. coli* strains (9). Many studies have demonstrated Lactobacillus and Bifidobacterium’s antioxidant activities that help defend against pathogen infection (10–12). *Lactobacillus rhamnosus* GG inhibited autophagy induced by *Salmonella enterica serovar* Infantis though promoting EGFR-mediated Akt activation (13). Several studies have analyzed probiotics for the treatment of gastrointestinal infections; however, few studies are more interested in the application of probiotics in breast. A study indicated that intestinal microbiota dysbiosis may be one of the causes of mastitis, and probiotics can improve the symptoms of mastitis (14). Meanwhile, our previous study showed that *Lactobacillus rhamnosus* GR-1 (LGR-1) reduced excessive NOD-like receptor family member pyrin domain-containing protein 3 (NLRP3) inflammasome activation induced *by E. coli*, thereby reducing interleukin-1 beta (IL-1β) secretion (15). However, the mechanism employed by LGR-1 to attenuate the NLRP3 signaling pathway activation in *E. coli*-induced bovine mastitis needs to be elucidated.

During a microbial infection, the host immune system gets activated. Increasing evidence has shown autophagy’s role in regulating this immune response and inflammation to resist microbial infection (16, 17). The deletion of autophagic protein Beclin-1 in macrophages isolated from the mouse lead to the activation of NLRP3 inflammasome and the increased secretion of IL-1β and interleukin-18 (IL-18) (18). Autophagy activation inhibited the secretion of IL-1β and enhanced the degradation of inflammasome (19). These findings indicated that autophagy negatively regulates inflammasome activation. Mitophagy, the selective autophagy of mitochondria, reduces the production of free radicals, adjusts the dynamic balance of mitochondria, and maintains cell survival (20). Although the correlation between mitophagy and NLRP3 inflammasome activation is unclear, mitochondria-derived reactive oxygen species (ROS) are known to participate in NLRP3 inflammasome activation (21). When mitophagy function is impaired, the damaged mitochondria-induced the excessive accumulation of ROS (mtROS) causes NLRP3 inflammasome activation and leads to an inflammatory cascade (22). NLRP3 inflammasome has been proved to play a key role in the host defense against microbial infections (23, 24). Studies have shown that NLRP3 can identify *Salmonella typhimurium* infections in macrophages and promote Caspase-1-dependent cell death and IL-1β production (25). Researchers also observed NLRP3 inflammasome activation induced by *E. coli* (15), *C. rodentium* (26) and *Staphylococcus aureus* (27) infection in macrophages. These studies indicated that the activation of NLRP3 inflammasome was very closely related to the interaction between host and bacteria. In our previous study, LGR-1 attenuate *E. coli*-induced NLRP3 inflammasomes activation, however, mechanism is not yet known. Moreover, during infection, *E. coli* decreases mitochondrial transmembrane potential, increase depolarization rate (28), and reactive oxygen species (ROS) levels (29) and subsequently results in cell apoptosis. However, the role of mitochondrial damage and mitophagy in *E. coli*-induced bovine mastitis is not known. The protective role of mitophagy pathway during LGR-1 action needs to be explored.

Therefore, we hypothesize that LGR-1 probiotic inhibits *E. coli*-induced cell apoptosis NLRP3 inflammasome assembly and by blocking ROS production and mediating mitophagy activation. In the present study, we examine the correlation between probiotics and mitophagy and its role in defense against the pathogen, specifically during coliform mastitis.

## Results

### LGR-1 Alleviates Mitophagy Inhibition in *E. coli*-Infected MAC-T Cells

**Figure 1:**
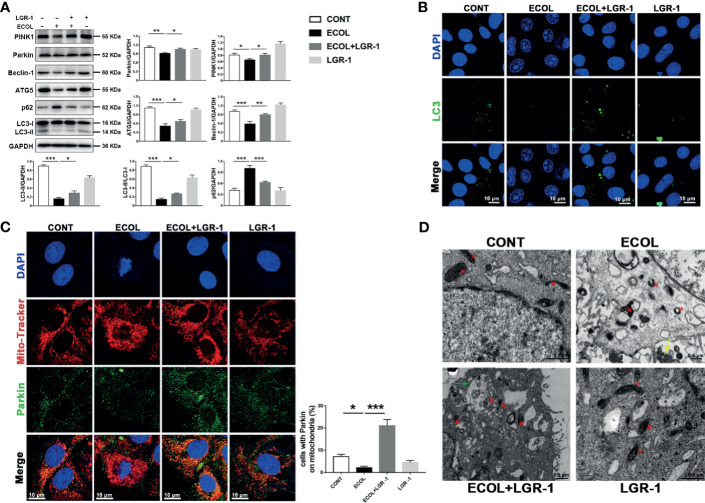
LGR-1 alleviates *Escherichia coli*-induced inhibition of mitophagy in MAC-T cells. **(A)** Western blot analysis of PINK1, Parkin, Beclin-1, ATG5, p62, and LC3. The lower right panel shows the protein quantification using ImageJ software (version 1.50). **(B)** Representative immunofluorescence images of LC3 (green). DAPI (Blue) stains the nucleus. Scale bar, 10 µm. **(C)** Representative images and quantification of immunofluorescence double-labelling Parkin (Green) and mitochondrial marker (MitoTracker, red). DAPI (Blue) stains the nucleus. Scale bar, 10 µm. **(D)** Representative TEM images of mitochondria and autophagosomes. Red M, mitochondria; Green arrows, autophagosome; Yellow arrows, *E coli*. Scale bar, 0.5 µm. Confluent MAC-T cell were pretreated with LGR-1 (10^5^ CFU/mL, MOI = 1) for 3 h, washed three times with PBS, and exposed to *E coli* (10^7^ CFU/mL, MOI = 66). Cells were analyzed 8 h after *E coli* infection. Data presented are mean ± SEM; n = 3. *p < 0.05, **p < 0.01, ***p < 0.001.

Autophagy is a host defense system that plays a vital role in resisting bacterial infections. First, we explored the correlation between *E. coli* infection and autophagy. After 8 h, *E. coli* infection reduced LC3II, ATG5, and Beclin1 expression levels and LC3II to LC3I ratio compared with the control, while LGR-1 pretreatment alleviated this reduction (**Figure 1A**). In contrast, *E. coli* infection increased p62 expression, a common autophagy substrate, indicating autophagic flux blockage, while LGR-1 pretreatment attenuated this increase (**Figure 1A**). Confocal laser scanning confirmed the inhibition of autophagy in MAC-T cells (**Figure 1B**). Besides, *E. coli* infection significantly decreased LC3 puncta while LGR-1 pretreatment increased LC3 puncta (**Figure 1B**). These results suggest that *E. coli* inhibits autophagy, and LGR-1 alleviates this inhibition. Interestingly, *E. coli* significantly reduced PINK1 and Parkin protein levels, and LGR-1 pretreatment prevented this reduction (**Figure 1A**). Furthermore, immunofluorescence revealed colocalized Parkin and MitoTracker in LGR-1 pretreated MAC-T cells, indicating the formation of mitophagosomes (**Figure 1C**). Consistent with the immunofluorescence results, TEM showed mitophagosomes in LGR-1 pretreated MAC-T cells (**Figure 1D**). TEM results also showed that *E. coli* can cause mitochondria swell, mitochondrial cristae disappear and vacuolization (**Figure 1D**). Collectively, our findings indicate PINK1/Parkin–mediated mitophagy was inhibited in MAC-T cells infected with *E. coli*.

**Figure 2:**
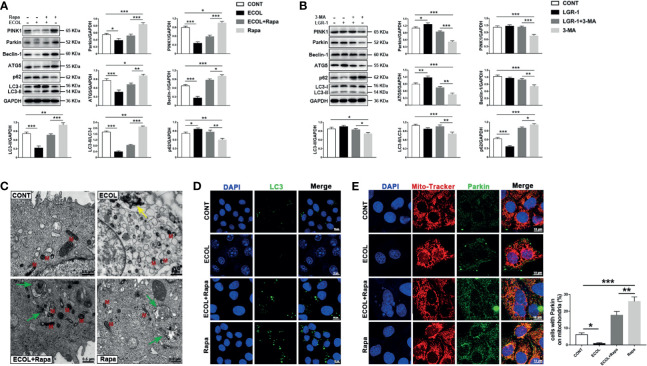
LGR-1 reduces the inhibitory effect of *Escherichia coli* on mitophagy in MAC-T cells. **(A, B)** Western blot analysis of PINK1, Parkin, Beclin-1, ATG5, p62, and LC3. The lower right panel shows the protein quantification using ImageJ software (version 1.50). **(C)** Representative TEM images of mitochondria and autophagosomes. Red M, mitochondrial; Green arrows, autophagosome; Yellow arrows, *E coli*. Scale bar, 0.5 µm and 1 µm. **(D)** Representative immunofluorescence images of LC3 (Green). DAPI (Blue) stains the nucleus. Scale bar, 10 µm. **(E)** Representative images and quantification of immunofluorescence double-labelling Parkin (green) and mitochondrial marker (MitoTracker, Red). DAPI (Blue, nucleus). Scale bar, 10 µm. MAC-T cells were pretreated with 3-methyladenine (3-MA, 5 mM) for 12 h and then treated with LGR-1 (MOI = 1) for 3 h or pretreated with rapamycin (Rapa, 2 μM) for 12 h and then treated with *E coli* (MOI = 66) for 8 (h) Data presented are mean ± SEM; n = 3. *p < 0.05, **p < 0.01, ***p < 0.001.

In order to further verify the relationship between LGR-1, *E. coli* and mitophagy, we used promoter and inhibitor for positive and negative verification respectively. MAC-T cells were incubated with 3-MA before exposure to LGR-1 and Rapa for 12 h before exposure to *E. coli*. Immunoblot analysis of PINK1, Parkin, p62, LC3, ATG5, and Beclin-1 showed that mitophagy was inhibited by 3-MA and activated by Rapa. The increased levels of PINK1, Parkin and LC3II proteins, proved that Rapa promoted mitophagy. The decreased levels of PINK1, Parkin and LC3II proteins, proved that 3-MA inhibited mitophagy. *E. coli* decreased PINK1, Parkin, p62, LC3, ATG5, and Beclin-1 expression levels and increased p62 expression level even with Rapa pretreatment (**Figure 2A**). On the contrary, LGR-1 increased PINK1, Parkin, p62, LC3, ATG5, and Beclin-1 expression levels and decreased p62 expression level even with 3-MA pretreatment (**Figure 2B**). Similarly, *E. coli* significantly reduced the number of LC3 puncta (**Figure 2D**). Furthermore, the colocalization of Parkin and MitoTracker was reduced (**Figure 2E**) after *E. coli* infection in cells pretreated with Rapa compared with Rapa control. MitoTracker staining showed mitochondria with a clear outline and a network structure in Rapa-pretreated MAC-T cells compared with the *E. coli* group (**Figure 2E**). TEM also showed mitophagosomes and normal mitochondria in Rapa-pretreated MAC-T cells before exposure to *E. coli* (**Figure 2C**). These findings suggest that the promotion of mitophagy in MAC-T cells reduced the mitochondrial damage by *E. coli* and LGR-1 may play a certain protective role by reducing the inhibition of *E. coli* on mitophagy.

### LGR-1 Activates Mitophagy to Suppress *E. coli*-Induced NLRP3 Inflammasome Activation and Apoptosis

**Figure 3:**
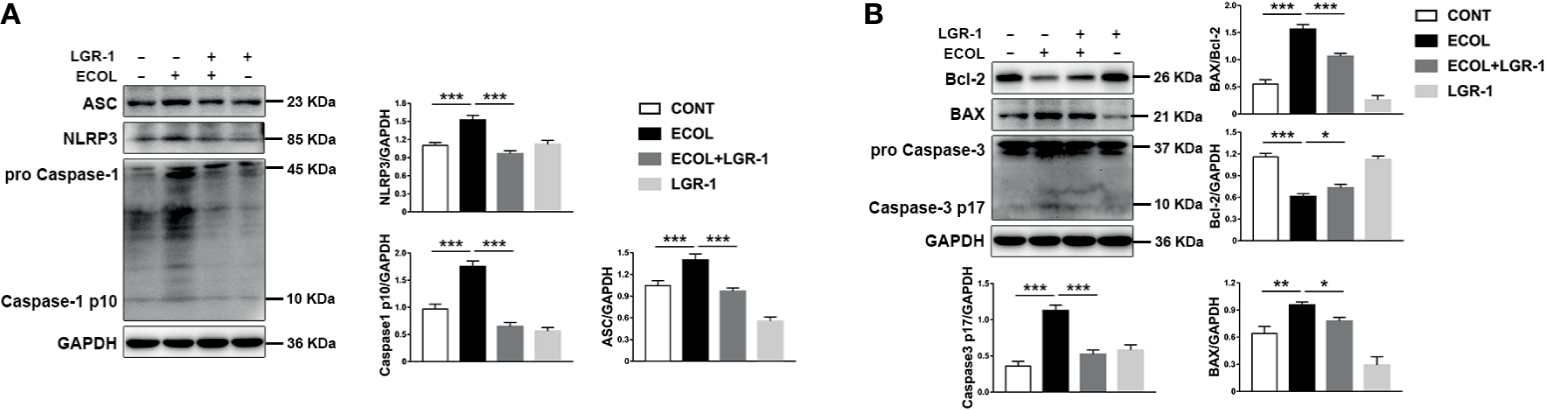
LGR-1 alleviates *Escherichia coli-*induced NLRP3 inflammasome activation and apoptosis in MAC-T cells. **(A, B)** Western blot analysis of ASC, NLRP3, Caspase-1, BAX, Bcl-2, and Caspase-3. The lower right panel shows the protein quantification using ImageJ software (version 1.50) Confluent MAC-T cell were pretreated with LGR-1 (10^5^ CFU/mL, MOI = 1) for 3 h, washed three times with PBS, and exposed to *E coli* (10^7^ CFU/mL, MOI = 66). Cells were analyzed 8 h after *E coli* infection. Data presented are mean ± SEM; n = 3. *p < 0.05, **p < 0.01, ***p < 0.001.

Further, we investigated the regulatory role of mitophagy on NLRP3 inflammasome and apoptosis. Immunoblot analysis showed an increase in NLRP3, ASC, and Caspase-1 p10, indicating NLRP3 inflammasome activation in cells infected with *E. coli*, and LGR-1 reduced this increase (**Figure 3A**). Chromatin deep staining and nuclear fragmentation, the apoptosis-related events, were observed after *E. coli* infection (**Figures 1B, C**). *E. coli* infection increased BAX and Caspase-3 p17 expression levels and BAX to Bcl-2 ratio and decreased Bcl-2 expression level; LGR-1 pretreatment reversed these phenomena (**Figure 3B**). These data suggest that LGR-1 can reduce the NLRP3 inflammasome activation and apoptosis induced by *E. coli*.

**Figure 4:**
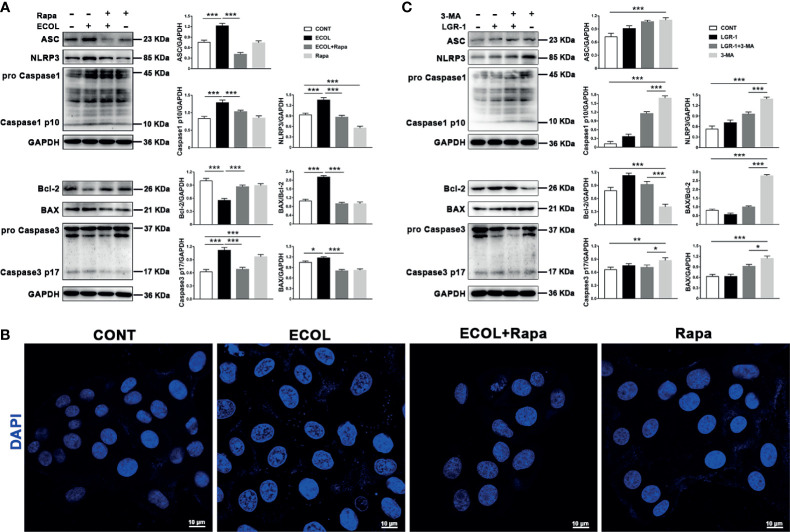
LGR-1 alleviates *Escherichia coli-*induced inhibition of mitophagy to suppress NLRP3 inflammasome activation and apoptosis in MAC-T cells. **(A, C)** Western blot analysis of ASC, NLRP3, Caspase-1, BAX, Bcl-2, and Caspase-3. The right panel shows the protein quantification by ImageJ software (version 1.50). **(B)** Cell apoptosis was detected by staining the nucleus with DAPI (Blue). Scale bar, 10 µm. MAC-T cells were pretreated with LGR-1 (MOI = 1) for 3 h and then treated with 3-methyladenine (3-MA, 5 mM) for 12 h or pretreated with rapamycin (Rapa, 2 μM) for 12 h and then treated with *E coli* (MOI = 66) for 8 (h) Data presented are mean ± SEM; n = 3. *p < 0.05, **p < 0.01, ***p < 0.001.

Autophagy negatively regulates NLRP3 inflammasome activation and apoptosis. In order to try to clarify the effect of *E. coli*/LGR-1 on mitophagy in MAC-T cells without considering other factors, and try to simulate the protective mechanism of LGR-1, Rapa and 3-MA were introduced. LGR-1 pretreatment prior to *E. coli* infection improved cell state by promoting mitophagy. Rapa displayed a potent inhibitory effect on NLRP3 and Caspase-1 activation in *E. coli* challenged cells (**Figure 4A**). In *E. coli*-infected MAC-T cells, Caspase-3 p17 and BAX expression levels and BAX to Bcl-2 ratio decreased and Bcl-2 expression level decreased by Rapa pretreatment (**Figure 4A**). Similarly, the state of the nucleus in Rapa-pretreated cells was better than in the cells infected with *E. coli* after Rapa pretreatment (**Figure 4B**). In the presence of the autophagy inhibitor 3-MA, NLRP3 inflammasome and apoptosis were activated, and LGR-1 pretreatment alleviated this situation (**Figure 4C**). These data indicate that activated mitophagy suppresses *E. coli*-induced NLRP3 inflammasome activation and apoptosis.

### LGR-1 Alleviates *E. coli*-Induced ROS Production and Mitochondrial Damage in MAC-T Cells

**Figure 5:**
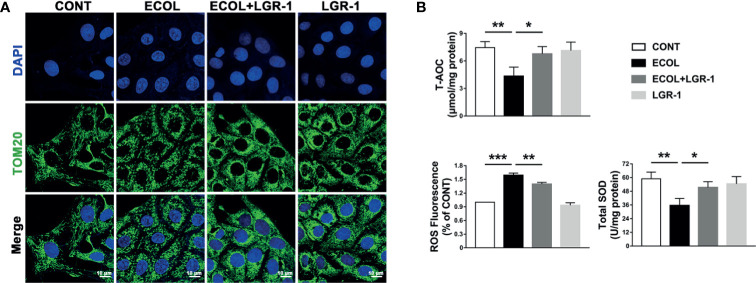
LGR-1 alleviates *Escherichia coli-*induced ROS production, mitochondrial damage in MAC-T cells. **(A)** Representative immunofluorescence images of TOM20 (Green). DAPI (Blue, nucleus). Scale bar, 10 µm. **(B)** T-AOC and SOD and ROS levels. Confluent MAC-T cell were pretreated with LGR-1 (10^5^ CFU/mL, MOI = 1) for 3 h, washed three times with PBS, and exposed to *E coli* (10^7^ CFU/mL, MOI = 66). Cells were analyzed 8 h after *E coli* infection. Data presented are mean ± SEM; n = 3. *p < 0.05, **p < 0.01, ***p < 0.001.

Furthermore, we analyzed the mitochondria in the MAC-T cells. TOM20 staining showed evenly distributed linear or short, rod-shaped, straight or curved mitochondria with clear outlines, forming a network structure throughout the cytoplasm (**Figure 5A**). Mitochondria were fragmented in the cells infected with *E. coli*, and LGR-1 pretreatment returned mitochondria to normal. TEM also showed severe mitochondrial damage, manifested as mitochondrial swelling, loss of cristae, and vacuolization, in cells infected with *E. coli*; LGR-1 alleviated the damage (**Figure 1D**). We further measured the T-AOC and ROS and SOD levels in the cells. *E. coli* decreased T-AOC and SOD level and increased ROS levels, and LGR-1 pretreatment alleviated these trends (**Figure 5B**). Collectively, our findings indicate that *E. coli* activates NLRP3 inflammasome and apoptosis by causing mitochondrial damage and oxidative stress and LGR-1 alleviates these effects.

### LGR-1 Eliminates *E. coli*-Induced ROS to Suppress NLRP3 Inflammasome Activation and Apoptosis

**Figure 6:**
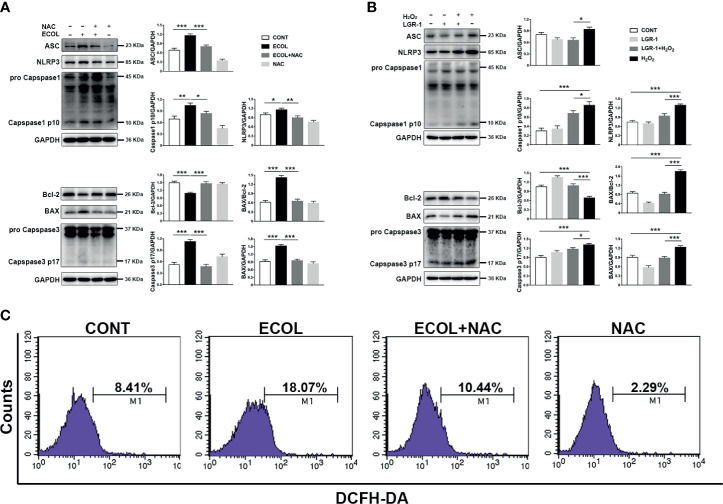
*Escherichia coli*-induced NLRP3 inflammasome activation and apoptosis requires ROS production in MAC-T cells. **(A, B)** Western blot analysis of ASC, NLRP3, Caspase-1, BAX, Bcl-2, and Caspase-3. The right panel shows the protein quantification using ImageJ software (version 1.50). **(C)** Flow cytometry analysis of ROS. M1 represents the proportion of cells stained with DCFH-DA. MAC-T cells were pretreated with *N-acetyl-L-cysteine* (NAC, 5 mM) for 2 h and then exposed to *E coli* (MOI = 66) for 8 h or pretreated with LGR-1 for 3 h and then exposed to hydrogen peroxide (H_2_O_2_, 0.5 mM) for 0.5 (h) Data presented are mean ± SEM; n = 3. *p < 0.05, **p < 0.01, ***p < 0.001.

*E. coli* induced mitochondrial damage led to the accumulation of ROS, which stimulate NLRP3 and apoptosis. MAC-T cells infected by *E. coli* showed high ROS levels (**Figure 5B**). Meanwhile, the ROS levels in *E. coli*-infected cells pretreated with the ROS scavenger-NAC were similar to those in the control cells (**Figure 6C**). Furthermore, LGR-1 pretreatment prevented ROS production in response to *E. coli* infection (**Figure 5B**), showing the effective ROS scavenging activity of LGR-1. The ROS scavenging effect of LGR-1 was further demonstrated in H_2_O_2_-induced MAC-T cells (**Figure 8D**). We tested the activation of NLRP3 inflammasome and apoptosis after adding NAC. Accompanying the decrease in ROS, NLRP3, Caspase-1 p10, and Caspase-3 p17 levels (**Figure 6A**) due to *E. coli* infection was mitigated by NAC, which indicated that *E. coli*-induced NLRP3 inflammasome and apoptosis activation required ROS production. Therefore, after NAC treatment, the ability of *E. coli* to induce ROS production is weakened, and ROS levels return to normal levels. The increase in NLRP3, ASC, Caspase-1 p10, BAX, and Caspase-3 p17 levels and BAX to Bcl-2 ratio and the decrease in Bcl-2 level in H_2_O_2_-treated cell suggest that H_2_O_2_ activates NLRP3 inflammasome and apoptosis by producing ROS (**Figure 6B**). Meanwhile, LGR-1 showed an effect similar to NAC; LGR-1 alleviated H_2_O_2_-induced NLRP3 inflammasome activation and apoptosis (**Figure 6B**). Flow cytometry showed less Annexin V-positive MAC-T cells after LGR-1 pretreatment compared with H_2_O_2_ (**Figure 8C**). Meanwhile, LGR-1 pretreatment decreased the ROS levels in cells compared with H_2_O_2_-induced cells (**Figure 8D**). Based on these data, we speculate that LGR-1 eliminates ROS to relieve *E. coli-*induced NLRP3 inflammasome activation and apoptosis.

### LGR-1 Inhibits NLRP3 Inflammasome Activity to Reduce *E. coli*-Induced Apoptosis

**Figure 7:**
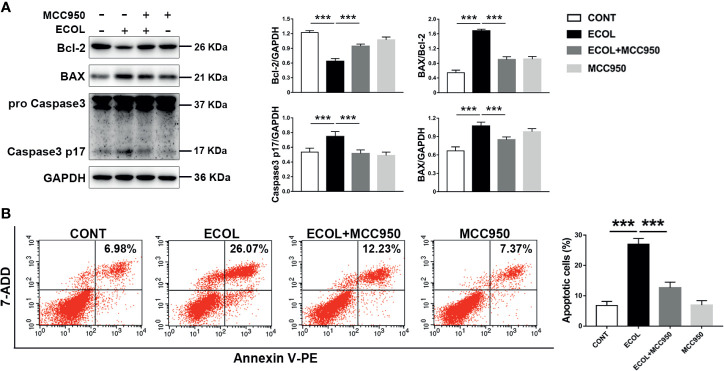
Inhibition of NLRP3 inflammasome activation reduces *Escherichia coli-*induced apoptosis in MAC-T cells. **(A)** Western blot analysis of BAX, Bcl-2, and Caspase-3. The right panel shows the protein quantification using ImageJ software (version 1.50). **(B)** Flow cytometry analysis of the percentage of apoptotic cells. MAC-T cells were pretreated with MCC950 (100 nM) for 0.5 h and then exposed to *E coli* (MOI = 66) for 8 (h) Data presented are mean ± SEM; n = 3. ***p < 0.001.

LGR-1 attenuated NLRP3 inflammasome activation and apoptosis induced by *E. coli*; therefore, we explored the relationship between NLRP3 inflammasomes and apoptosis. Immunoblot analysis showed an increase in the pro-apoptotic protein (BAX and Caspase3) and decrease in the anti-apoptotic protein (Bcl-2) with *E. coli* exposure (**Figures 3B**, **4A**, **6A** and **7A**). MCC950, an NLRP3 inflammasome inhibitor, was used to pretreat MAC-T cells before exposure to *E. coli* to inhibit NLRP3 inflammasome activation and explore the correlation between NLRP3 inflammasome and *E. coli*-induced apoptosis. MCC950 abolished *E. coli*-induced apoptosis, which was demonstrated by the decrease in BAX, BAX/Bcl-2 ratio and Caspase-3 p17 level and an increased in Bcl-2 level in immunoblot analysis (**Figure 7A**). Flow cytometry also showed less Annexin V-positive MAC-T cells after MCC950 pretreatment (**Figure 7B**). Collectively, our findings indicate that LGR-1 inhibits NLRP3 inflammasome activity to protect MAC-T cells from *E. coli*-induced apoptosis.

### Silencing PINK1 Blocks the Inhibitory Effect of LGR-1 on ROS, NLRP3 Inflammasome, and Apoptosis

**Figure 8:**
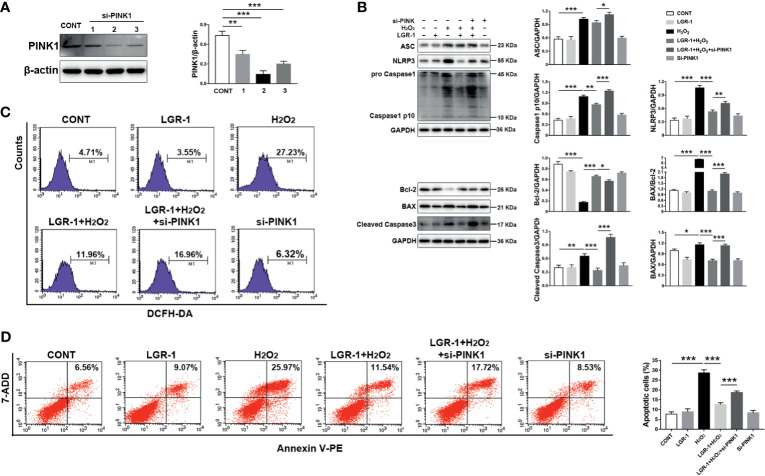
Silencing PINK1 blocks the inhibitory effect of LGR-1 on *Escherichia coli-*induced ROS release, NLRP3 inflammasome activation, and apoptosis in MAC-T cells. **(A)** Western blot analysis of PINK1 in MAC-T cells after PINK1silencing (si-PINK1). The right panel shows the protein quantification using ImageJ software (version 1.50). **(B)** Western blot analysis ASC, NLRP3, Caspase-1, BAX, Bcl-2, and Caspase-3 in MAC-T cells after PINK1silencing (si-PINK1). The right panel shows the protein quantification using ImageJ software (version 1.50). **(C)** Flow cytometry analysis of ROS. M1 represents the proportion of cells stained with DCFH-DA. **(D)** Flow cytometry analysis of the percentage of apoptotic cells. The si-PINK1-MAC-T cells pretreated with LGR-1 for 3 h and then exposed to hydrogen peroxide (H_2_O_2_, ROS inducer; 0.5 mM) for 0.5 (h) Data presented are mean ± SEM; n = 3. *p < 0.05, **p < 0.01, ***p < 0.001.

To verify whether LGR-1 eliminates ROS production through mitophagy mediated by PINK1/Parkin to inhibit the activation of NLRP3 inflammasome and apoptosis, we applied siRNA interference technology to knock down the PINK1 protein expression in MAC-T cells. Immunoblot analysis confirmed that siRNA effectively suppressed PINK1 expression (**Figure 8A**). We further added H_2_O_2_ as a positive control to increase ROS production and activate NLRP3 inflammasome and apoptosis. LGR-1 could not reduce H_2_O_2_-induced NLRP3, ASC, and Caspase-1 p10 levels after PINK1 was knocked down (**Figure 8B**). Besides, LGR-1 could not alleviate H_2_O_2_-apoptosis, manifested by the increase in BAX, BAX/Bcl-2 ratio, and cleaved-Caspase-3, and the decrease in Bcl-2 (**Figure 8B**). Knocking down PINK1 inhibited the ROS scavenging effect of LGR-1 (**Figure 8C**). Flow cytometry showed more Annexin V-positive MAC-T cells after silencing PINK1 compared with the H_2_O_2_-induced cells pretreated with LGR-1 (**Figure 8D**). These results indicate that LGR-1 exerts a beneficial effect through PINK/Parkin-mediated mitophagy.

### LGR-1 Alleviates *E. coli*-Induced Mitophagy Inhibition, NLRP3 Inflammasome Activation, and Apoptosis in Mice Mammary Gland

**Figure 9:**
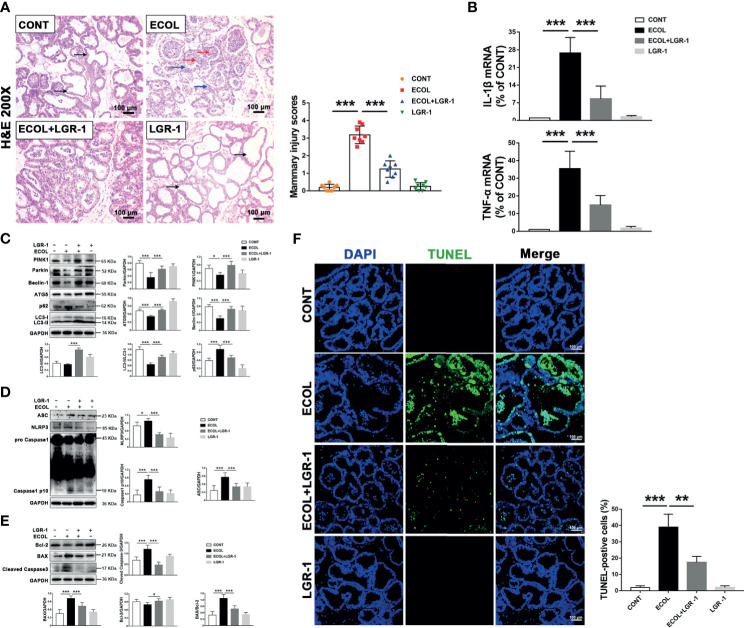
LGR-1 alleviates *E coli*-induced mitophagy inhibition, NLRP3 inflammasome activation, and apoptosis in mice mammary gland. **(A)** Mammary gland tissue sections were stained with H & **(E)** The black arrow was the normal tissues. The red arrow was the infiltration of inflammatory cells. The blue arrow was the hyperplastic of alveolar wall. The scoring criteria are varying from 0 points to 4 points in no injury, mild injury, moderate injury, severe injury, and extreme injury, respectively. Scale bar, 100 µm. **(B)** The mRNA levels of IL-1β and TNF-α in mammary tissue homogenate. **(C, D, E)** Western blot analysis of PINK1, Parkin, Beclin-1, ATG5, p62, LC3, ASC, NLRP3, Caspase-1, BAX, Bcl-2, and cleaved Caspase-3. The right and lower panel shows the protein quantification using ImageJ software (version 1.50). **(F)** Apoptosis was accessed by TUNEL staining of mammary and quantification of TUNEL-positive cells. DAPI (Blue, nucleus). Scale bar, 100 µm. LGR-1 was given by gavage (2.5 × 10^8^ CFU/200 μL saline, 7 consecutive days, once a day), and then *E coli* (1 × 10^6^ CFU/30 μL saline) was injected into mammary duct to establish a mouse mastitis model (24 hours later, the mice were euthanized and mammary tissue was collected). Data presented are mean ± SEM; n = 8. *p < 0.05, ***p < 0.001.

In order to verify whether LGR-1 has obvious protective effect *in vivo*, we successfully established a mouse mastitis model. First, we observed the morphological and histological changes of mammary gland tissue by H&E staining. There was no significant change in the mammary glands of the CONT group and LGR-1 group. In ECOL group, the wall of mammary acinus was obviously thickened, the stroma was congested, and the acinus was filled with inflammatory cells (**Figure 9A**). And LGR-1 could significantly improve the histopathological changes caused by *E. coli*. Further detection of cytokines expression showed that *E. coli* could significantly increase IL-1 β and TNF- α, whereas LGR-1 can inhibit the production of these inflammatory factors in the breast (**Figure 9B**). Then, in order to detect whether LGR-1 can affect mitophagy, NLRP3 inflammasome and apoptosis, we detected the changes of related proteins by Western blotting. In line with the results of *in vitro*, *E. coli* can inhibit mitophagy related protein (PINK1, Parkin, ATG5, Beclin-1 and LC3II/LC3I), increase the expression of NLRP3 inflammasome (NLRP3, ASC and Caspase1 p10), and cause apoptosis, while LGR-1 can reduce these effects of *E. coli* (**Figures 9C–E**). Finally, TUNEL staining was performed to further explore the effect on apoptosis. The results showed that *E. coli* induced the accumulation of TUNEL-positive cells in the epithelial cells of breast tissue, while LGR-1 could significantly reduce the proportion of TUNEL-positive cells (**Figure 9F**).

## Discussion

**Figure 10:**
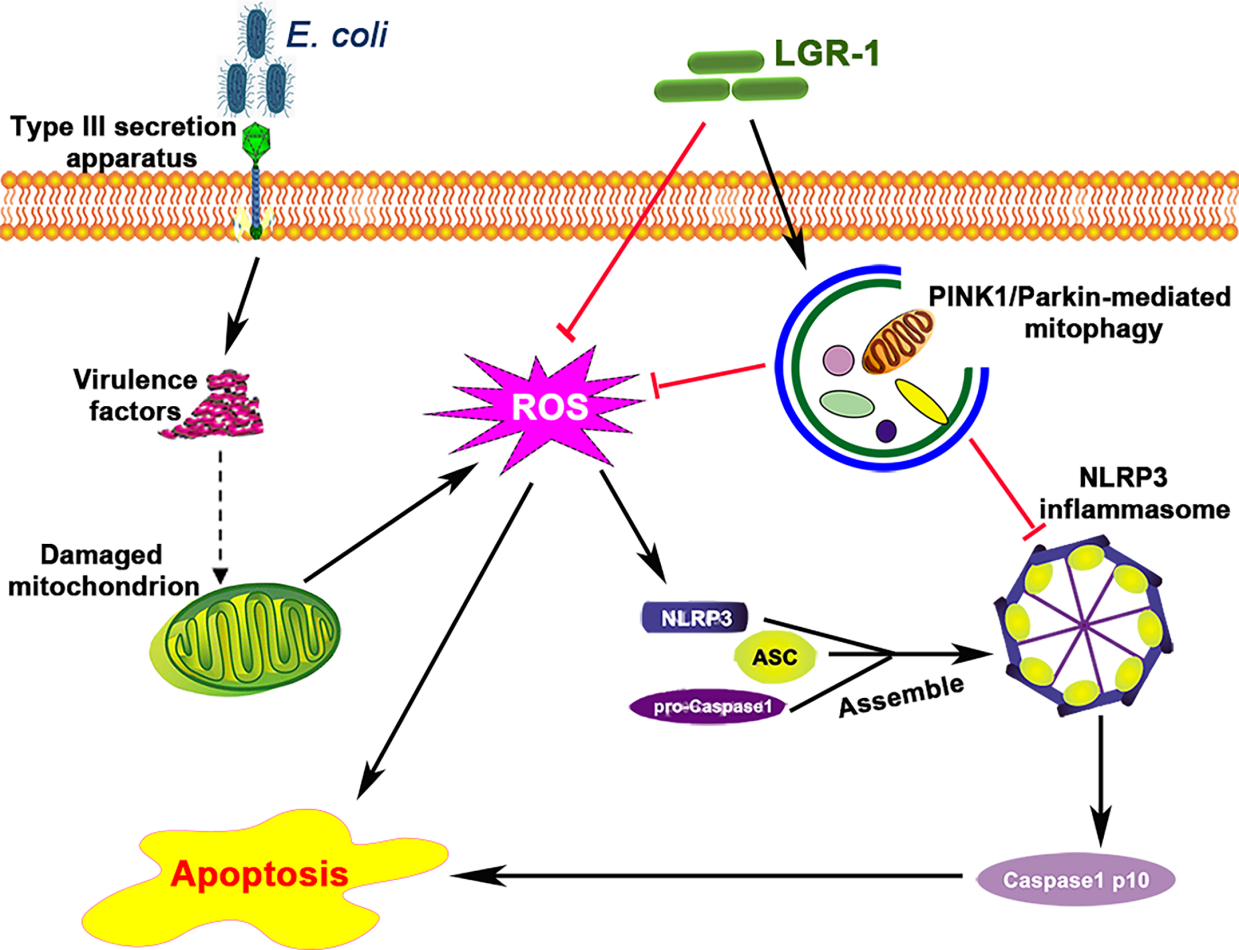
*Lactobacillus rhamnosus* GR-1 prevents *Escherichia coli*-induced cell apoptosis through PINK1/Parkin-mediated mitophagy by inhibiting ROS production and NLRP3 inflammasome activation. *E. coli* secretes virulence factors *via* the type III secretion system and causes mitochondrial dysfunction. The damaged mitochondrion produces excessive reactive oxidative species (ROS), which induces NLRP3 inflammasome activation and subsequently causes apoptosis. LGR-1 has anti-ROS function and inhibits ROS production. LGR-1 pretreatment induces mitophagy that eliminates ROS and inhibits NLRP3 inflammasome activation. The solid arrows indicate demonstrated effects, the dashed arrow indicates the potential effects, and the red lines indicate negative effects.

*E. coli* is the main causative agent of clinical mastitis in dairy cattle, which is difficult to cure and results in huge economic losses. During bacterial infection, many pathogen-associated molecular patterns (30), such as type III secretion system and lipopolysaccharides, get expressed on the cell wall, which activate inflammasomes, including NLRP3 inflammasome. The NLRP3 inflammasome has been known to play a role in many inflammatory diseases (31, 32), and its excessive activation induces intestinal and breast inflammation and tissue damage (3, 33). Consistent with the previous studies (34), our study confirms that *E. coli* can activate NLRP3 inflammasome in mammary epithelial cells also (**Figure 1**). Besides, mitophagy, a host defense mechanism, prevents excessive activation of NLRP3 inflammasome (18, 21, 35). Therefore, a balance between these two is vital to prevent microbial response and maintain immunity and health. However, the mechanism *via* which mitophagy regulates the NLRP3 inflammasome in breast diseases, especially during mastitis, remains unknown. Our study for the first time showed that mitophagy mediated by PINK1/Parkin was inhibited in *E. coli*-induced mastitis (**Figure 10**).

Probiotics, as an alternative to antibiotics, have attracted scientific interest in mastitis treatment. *Lactobacillus rhamnosus* induced STAT3 and JNK activation through granulocyte colony-stimulating factor and consequently inhibited tumor necrosis factor production in lipopolysaccharide-or *E. coli*-activated macrophages (36). Similarly, *Lactobacillus johnsonii* L531 inhibited *Salmonella* Infantis-induced activation of NLRP3 inflammasome (33). Meanwhile, our previous study showed that LGR-1 attenuated NLRP3 inflammasome activation induced by *E. coli*. These studies collectively indicate the beneficial effects of probiotics; however, the specific mechanism of LGR-1 in inhibiting NLRP3 inflammation activation to prevent mastitis remains unclear. We found that LGR-1 inhibited pathogen infection by triggering mitophagy to inhibit ROS production, NLRP3 inflammasome activation and apoptosis in breast epithelial cells (**Figure 10**), which provides an understanding of the mechanism *via* which probiotics demonstrate antimicrobial effects and maintain breast health. A study showed that blocking NLRP3 activation provided protection against lethal influenza A virus (IAV) (37). Consistent with that study, we found that LGR-1 diminished *E. coli*-induced NLRP3 assembly and pro-Caspase-1 cleavage. Thus, the study confirms the selective inhibition of NLRP3 inflammasome by LGR-1 to rescue cellular damage caused by pathogens.

Recently, many studies have proven the key role of mitochondria in innate immune response (38, 39). However, mitochondrial damage can lead to the accumulation of ROS, which can activate NLRP3 inflammasome and apoptosis (18, 40). In MAC-T cells, *E. coli* infection led to massive ROS release and severe mitochondrial damage (**Figures 1**, **2** and **5**). *E. coli* induced chromatic agglutination and karyopyknosis, increased BAX and cleaved-Caspase-3 expression levels, and decreased Bcl-2 expression level, indicating apoptosis (**Figure 3**). In view of this, we speculate that ROS is necessary for *E. coli* to induce NLRP3 inflammasome activation and apoptosis. Therefore, we explored the importance of ROS for *E. coli*-induced NLRP3 inflammasome activation and apoptosis by treating cells with NAC (a ROS scavenger). The results showed that NAC prevented *E. coli*-induced the activation of NLRP3 inflammasome and apoptosis in MAC-T cells (**Figure 6A**). The observation indicated that the activation of *E. coli-*induced NLRP3 requires ROS release. Parallelly, LGR-1 can attenuate ROS activation and apoptosis induced by *E. coli* (**Figures 3** and **5**). Thus, we speculate that lgr-1 has the ability to clear ROS. To verify the role of LGR-1 in reducing ROS production, H_2_O_2_, a ROS stimulant, was introduced to the cell culture. As shown in **Figure 6B**, LGR-1 alleviated the increase of NLRP3 protein and pro-apoptotic protein, and the decrease of anti-apoptotic protein induced by H_2_O_2,_ suggesting the anti-ROS, anti-inflammatory, and antiapoptotic effects of LGR-1.

Furthermore, mitochondrial damage was associated with NLRP3 inflammasome activation and apoptosis induced by *E. coli*. Widespread mitochondrial damage was detected when autophagy was inhibited. Therefore, eliminating the damaged mitochondria by autophagy is necessary to prevent excessive activation of NLRP3 inflammasome (18, 21, 35). Mitophagy, a selective autophagy in mitochondria, is a crucial process for regulating NLRP3 inflammasome activation by removing damaged mitochondria. PINK1/Parkin pathway has been reported to regulate mitophagy. In normal conditions, PINK1 is imported into mitochondria, anchored in inner mitochondrial membrane, and degraded by mitochondrial proteases. However, when mitochondria are damaged, PINK1 does not get imported into inner mitochondrial membrane, but aggregates on outer mitochondrial membrane (41), recruits and activates Parkin, binds with LC3 on the autophagosome, and promotes damaged mitochondria degradation (39). In a variety of inflammatory diseases, moderate regulation of mitophagy is important for maintaining mitochondrial homeostasis. In autophagy/mitophagy-deficient macrophages, NLRP3 activators increased the accumulation of damaged mitochondria, accompanied by ROS production. Consistent with this, *E. coli* reduced PINK1, Parkin, LC3II, ATG5, and Beclin-1 expression levels and Mito-Tracker/Parkin colocalization and increased p62 expression level, ROS production (**Figures 1**, **2**, and **5**), NLRP3 inflammasome activation, and apoptosis (**Figures 3** and **4**) in MAC-T cell. These findings together suggest that *E. coli* can inhibits mitophagy. However, LGR-1 can reverse the inhibitory effect of *E. coli* on mitophagy. In order to verify the regulatory effects of *E. coli* and LGR-1 on mitophagy, mitophagy promoter and inhibitor were introduced for verification. As shown in **Figure 4**, the results were consistent with the above speculation. Hence, LGR-1 alleviated the inhibitory effect of *E. coli* on mitophagy and weakened NLRP3 inflammasome activation and apoptosis in MAC-T cells.

Studies have proven the protective role of mitophagy mediated by PINK1/Parkin in apoptosis (42, 43). And study has found that increasing the expression of SESN2 to promote mitophagy can protect the host from sepsis (44). However, the specific pathway of mitophagy that regulates apoptosis is not yet reported in in mastitis induced by *E. coli*. This study elucidates the protective role of mitophagy mediated by PINK1/Parkin under *E. coli*-induced mastitis in MAC-T cells using 3-MA and Rapa (**Figures 2** and **4**). Besides, pretreatment of cells with NAC (ROS scavenger) and MCC950 (NLRP3 inhibitor) showed that apoptosis was inhibited through decreased ROS production and NLRP3 inflammasome activation in MAC-T cells (**Figures 6** and **7**). Then, in order to verify the importance of the PINK1/Parkin pathway in the protection of LGR-1, the PINK1 protein was knocked down. When the PINK1 protein is knocked down, LGR-1 cannot attenuate the accumulation of ROS, the NLRP3 inflammasome activation and apoptosis, thus cannot exert its protective effect. (**Figure 8**). Our findings collectively indicate that PINK1/Parkin-mediated mitophagy is one of the self-limiting pathways *via* which LGR-1 protects cells from excessive inflammation and apoptosis.

All of the above are our application of breast epithelial cells as an *in vitro* infection model to explore the protective effect of LGR-1 in mastitis. However, whether LGR-1 still has a protective effect *in vivo* is still unknown. Therefore, we established a mouse model of mastitis to explore the effects of LGR-1 *in vivo*. These results indicated that LGR-1 alleviated the inhibitory effect of *E. coli* on mitophagy and reduced the apoptosis and the increase of inflammatory factors induced by *E. coli* in mouse mastitis (**Figure 9**). Consequently, these data indicate that LGR-1 pretreatment has a preventive and protective effect in *E. coli*-induced mastitis (**Figure 9**). It has been reported that probiotics such as *clostridium tyrobutyricum* can significantly reduce the symptoms of mastitis induced by *Staphylococcus aureus*, which may be related to changes in gut microbiota and short-chain fatty acids in the intestine (14). We also used the same way of intragastric administration of probiotics, which may also change the state of gut microbiota in mice, and increase the content of probiotics such as lactic acid bacteria and short-chain fatty acids, so as to prevent mastitis. However, the application and effect of LGR-1 as a probiotic in bovine need to be further explored.

In summary, *E. coli* inhibited mitophagy and enhanced NLRP3 inflammasome activation and apoptosis, both *in vivo* and *vitro*. LGR-1 pretreatment induced PINK1/Parkin-mediated mitophagy, cleared the damaged mitochondria, and reduced ROS production, NLRP3 inflammasome activation and apoptosis under *E. coli* infection. These findings deepen understanding of probiotics’ immune protection and contribute to its application in bovine mastitis prevention and treatment. However, the protective mechanisms in bovine and the probiotic administration route need to be investigated. To conclude, our study suggests that therapeutic strategies aiming at the upregulation of mitophagy under *E. coli*-induced mastitis may preserve mitochondrial function in bovine mastitis.

## Materials and Methods

### Animals

Thirty-two females (8-10 week old) specific-pathogen-free pregnant Crl: CD1 (ICR) mice were purchased from Charles River (Beijing, China). Mice were reared in a sterile environment with 12h light and dark cycle. and ad libitum access to food and water.

### Ethics Statement

This study is approved by the Animal Ethics Committee of China Agricultural University, and all animal care and experimental procedures are under the supervision of this committee.

### Establishment of Mouse Mastitis Model

In order to verify the protective effect of LGR-1 on *E. coli* induced mastitis, we established a mouse mastitis model. This mouse mastitis model was induced by *E. coli* as previously described (14). In short, offspring were removed 4h before intramammary inoculation. The mice were anesthetized with Zoletil 50 (55mgkg, WK001, Virbac, France) and placed in supine position under the stereoscope. Disinfect the fourth pair of mammary glands and expose the mammary duct by cutting the tip of the nipple. *E. coli* dissolved in 30 µL physiologic saline was slowly intraductal injected through a 100-µL syringe with a 30-gauge blunt needle. Four groups (n = 8 per group) of mice were allocated: (1) a negative control group (CONT group); (2) *E. coli* group (ECOL group); (3) *E. coli* + LGR-1 group (ECOL + LGR-1 group); (4) LGR-1 group (LGR-1 group). Before *E. coli* infection, mice in the ECOL + LGR-1 and LGR-1 group were inoculated with LGR-1 (2.5 × 10^8^ CFU/200 μL saline) by oral gavage for 7 consecutive days, once a day; mice in the CONT and ECOL groups were administered an equal volume of sterile physiologic saline at 10:00 AM daily. At 10:00 AM on Day 8, ECOL and ECOL + LGR-1 group mice were intraductal injected *E. coli* (1 × 10^6^ CFU/30 μL saline), whereas mice in the CONT and LGR-1 group received equal volume of sterile physiologic saline. After 24 hours of *E. coli* infection, all mice were euthanized and the mammary glands tissue were collected and frozen at -80 °C until use.

### Bacterial Strains and Growth Conditions

*Lactobacillus rhamnosus* GR-1 (LGR-1, ATCC 55826; American Type Culture Collection, Manassas, VA, United States) was grown in De Man, Rogosa, and Sharpe (MRS) broth (Oxoid, Hampshire, United Kingdom) under microaerophilic conditions at 37°C for 24 h. After overnight incubation, LGR-1 was diluted (1:1000) in fresh MRS broth and grown for approximately 12 h until mid-log phase (OD600 = 0.6).

*Escherichia coli* strain (serotype O111:K58, CVCC1450, the China Institute of Veterinary Drug Center, Beijing, China) was grown in Luria–Bertani (LB) broth (Oxoid, Hampshire, United Kingdom). After overnight incubation, *E. coli* was diluted (1:1000) in fresh LB at 37°C for 8h and 200rpm until mid-log phase (OD600 = 0.6).

### Cell Culture and Infection

MAC-T cells (a kind gift from Dr. Ying Yu in China Agricultural University) were seeded in 6- or 12-well plates (3×10^5^ and 3×10^4^ cells per well, respectively) and cultured in DMEM/Ham’s F-12 (1:1) (GE Healthcare Life Sciences HyClone Laboratories, Utah, USA) supplemented with 10% FBS (ThermoFish Scientific, Rockford, USA) and penicillin (100 Units/mL)/streptomycin (100 μg/mL) at 37°C in a 5% CO_2_ incubator for 24 h. MAC-T cells were pretreated with LGR-1 (10^5^ CFU/mL; multiplicity of infection, MOI = 1) for 3 h. Then cells were washed three times with PBS and exposed to *E. coli* (10^7^ CFU/mL, MOI = 66). After 8 h, MAC-T cells were collected for further analysis.

### Drug Treatments

MAC-T cells were treated as follows to verify the role of autophagy in LGR-1 defense against *E. coli* infection: MAC-T cells were (1) pretreated with 3-methyladenine (3-MA, 5 mM, S2767, autophagy inhibitor from Selleck Chemicals, Houston, USA) for 12 h washed three times with PBS, and treated with LGR-1 (MOI = 1) for 3 h, or (2) pretreated with rapamycin (Rapa, 2 μM, S1039, autophagy activator from Selleck Chemicals, Houston, USA) for 12 h, washed three times with PBS, and challenge with *E. coli* (MOI = 66) for 8 h.

MAC-T cells were treated as follows to verify whether ROS is the target of LGR-1 in the defense against *E. coli* infection: MAC-T cells were (1) pretreated with N-acetyl-L-cysteine (NAC, 5 mM, S0077, ROS scavenger from Beyotime Biotechnology, Shanghai, China) for 2 h, washed three times with PBS, and exposed to *E. coli* (MOI = 66) for 8 h or (2) pretreated with LGR-1 for 3 h, washed three times with PBS, and exposed to hydrogen peroxide (H_2_O_2_, ROS inducer; 0.5 mM) for 0.5 h.

MAC-T cells were treated as follows to verify the correlation between NLRP3 inflammasome activation and cell apoptosis during *E. coli* infection: MAC-T cells were pretreated with MCC950 (100 nM, HY-12815A, NLRP3 inhibitor from MedChemExpress, New Jersey, USA) for 0.5 h, washed three times with PBS, and exposed to *E. coli* (MOI = 66) for 8 h.

In this study, MAC-T cells in all control groups were not treated, only washed with PBS synchronously. Both untreated and treated cells were collected for protein analysis.

### Histopathologic Scoring

The mammary tissues were fixed with 4% paraformaldehyde for at least 24h. The paraffin embedded tissues were sliced into 3 μm thick slices and stained with hematoxylin and eosin (H&E) staining to observe the pathological changes. The level of mammary inflammation was scored as described previously (45).

### RNA Interference

To verify the importance of PINK1/Parkin mediated-mitophagy pathway in the protection of LGR-1, we performed gene silencing to knocked down PINK. MAC-T cells were seeded in 24 well plates. When the cells grew to 60%-80% confluence, PINK1-siRNA (si-PINK1, sense 5’-GGAGCGGUCACUGACAGAATT-3’; antisense 5’-UUCUGUCAGUGACCGCUCCTT-3’, GenePharma, Suzhou, China) diluted with Lipofectamine™ RNAiMAX (13778075, a transfection reagent from ThermoFish Scientific, Rockford, USA) was transfected into the cells. At 5h after transfection, media were removed, supplemented with DMEM and incubated at 37°C for 48 h.

### Transmission Electron Microscopy

At 8 h after *E. coli* challenged, MAC-T cells were harvested and fixed in 3% glutaraldehyde (pH = 7.4) for 48 h. Samples were treated following the standard TEM procedure (15, 46).

### Immunofluorescence

MAC-T cells were cultivated on cell climbing sheets for staining and treated as described above (47). For labeling the mitochondria, Mito-Tracker Red CMXRos (CMXRos, C1035, Beyotime Biotechnology, Shanghai, China) was added to living MAC-T cells at 37°C for 30 min. The treated MAC-T cells were fixed with 4% paraformaldehyde for 10 min, then incubated with 1% Triton-X-100 (T8787, Sigma-Aldrich, St. Louis, USA) for 15 min at room temperature to permeate the cell membrane, and blocked with 2% bovine serum albumin for 1.5 h at room temperature. The cells were incubated with anti-Parkin (1:200, 14060-1-AP, Proteintech Group Inc, Rosemont, IL 60018, USA) or anti-TOM 20 (1:300, 11802-1-AP, Proteintech Group Inc), at 4°C overnight. Then cells were incubated with Alexa Fluor 488 goat anti-rabbit secondary antibody (Beyotime Biotechnology, Shanghai, China) at room temperature for 1 h. DAPI (C0060, Solarbio Science&Technology Co., Ltd, Beijng, China) was used to stain cell nuclei. The cells were observed and images captured under a confocal laser scanning microscope (Nikon A1); MitoTracker Red CMXRos was detected at 555 nm, and secondary antibodies at their corresponding wavelengths (488 nm/555 nm).

### Detection of Total Anti-Oxidation Capacity and Superoxide Dismutase Level

The T-AOC (S0121, Beyotime Biotechnology, Shanghai, China) and SOD (S0101S, Beyotime Biotechnology, Shanghai, China) level were determined using the commercial kits, following the manufacturers’ instructions.

### Measurement of Intracellular ROS

Intracellular ROS levels were measured by the 2,7-dichlorofluorescein diacetate (DCFH-DA, S0033S, cell-permeable fluorescent probe from Beyotime Biotechnology, Shanghai, China). Fluorescence was directly assessed by a fluorescence plate reader (BioTek Synergy H1). All the values were normalized using control. Flow cytometry was performed using a FACS Calibur system (BD Biosciences), and data were analyzed using FlowJO software (version 10.0.7).

### Quantitative Real-Time PCR

Total RNA was extracted from mice mammary tissue by using RNAiso Plus (9108, TaKaRa, Japan), and RNA transcription was performed adopting the PrimeScriptTM RT Reagent Kit (RR047A, TaKaRa, Japan). Quantitative real-time RT-PCR was performed through using a SYBR Green PCR Master Mix (LS2062, Promega, USA). The mRNA expression of IL-1β and TNF-α was normalized to the mRNA expression of β-actin. The primer sequences are demonstrated in **Table S1** and the gene expression levels were analyzed with the 2^−ΔΔCT^.

### Western Blotting

MAC-T cells were lysed in RIPA buffer containing a protease/phosphatase inhibitor cocktail on ice for 30 min.). Protein (equal amounts, 20 µg) were loaded on 10% or 12% SDS-polyacrylamide gels and transferred to polyvinylidene difluoride (PVDF) membranes (Roche). After blocking with 5% skim milk at 37°C for 1 h and the membranes were incubated with the following primary antibodies at 4°C overnight: anti-LC3 I/II (1:1000, #4108) and anti-cleaved-Caspase-3 (1:1000, #9664) from Cell Signaling Technology (Danvers, USA); anti-ATG5 (1:750, 10181-2-AP), anti-Beclin-1 (1:1000, 11036-1-AP), anti-p62/SQSTM1 (1:1000, 18420-1-AP), anti-ASC (1:1000, 10500-1-AP), anti-BAX (1:1000, 50599-2-Ig), anti-Bcl-2 (1:1000, 12789-1-AP), anti-Caspase-3 (1:1000), anti-PINK1 (1:1000, 23274-1-AP), anti-Parkin (1:1000, 14060-1-AP), anti-GAPDH (1:5000, 60004-1-AP) and anti-β-Actin (1:5000, 60008-1-AP) from Proteintech Group Inc (Rosemont, IL 60018, USA); anti-NLRP3 (1:500, AF2155) from Beyotime Biotechnology (Shanghai, China) and anti-Caspase-1 (1:1000, ab179515) from Abcam (Cambridge, UK). The immunoreactive bands were visualized with an ECL detection system (Tanon 6200 chemiluminescence imaging workstation, Tanon Science & Technology Co., Ltd. Shanghai, China). The protein bands were quantified by densitometry using ImageJ software (version 1.50).

### Flow Cytometry Assessment of Apoptosis

Cell apoptosis was measured by the Annexin V-PE/7-AAD apoptosis detection kit (A213-01, Vazyme Biotech, Nanjing, China). MAC-T cells were harvested, and softly re-suspended in 1× binding buffer (100 μL). Then Annexin V-PE (5 μL) and 7-ADD (5 μL) were added to each group and incubated in the dark for 15 min. Approximately 10,000 or 20,000 cells from each group were used to analyze on a FACS Calibur system (BD Biosciences) and evaluated with the FlowJo software (version 10.0.7).

### TUNEL Assay

The paraffin embedded tissues were sliced into 3 μm thick slices, dewaxed and dehydrated, and then TUNEL staining (A112, Vazyme, Nanjing, China) was performed according to the manufacturer’s instructions. Briefly, the slices were washed with PBS and reacted with TdT enzyme/buffer at 37°C for 1 h, followed by DAPI (C0060, Solarbio Science&Technology Co., Ltd, Beijng, China) staining at room temperature for 5 min. Then the slices were observed and images were captured at x400 magnification using a confocal laser scanning microscope (Nikon A1).

### Data Analysis

Using Prism 7 (GraphPad) to perform statistical analysis. Qualitative data were expressed as means ± standard error of the mean (SEM; n = 3 or 6). One-way analysis of variance (ANOVA) was applied to analyze statistically significant differences at *p* < 0.05, followed by Tukey’s test.

## Data Availability Statement

The original contributions presented in the study are included in the article/**Supplementary Material**. Further inquiries can be directed to the corresponding author.

## Ethics Statement

All animals were treated in strict accordance with the Guidelines for Laboratory Animal Use and Care from the Chinese Center for Disease Control and Prevention and the Rules for Medical Laboratory Animals (1998) from the Chinese Ministry of Health, under protocol CAU20161016- 1, which was approved by the Animal Ethics Committee of China Agricultural University.

## Author Contributions

YL: Conceptualization, methodology, validation, formal analysis, investigation, writing-original draft, supervision, and project administration. YZ: Methodology and funding acquisition. BC: Methodology and validation. NL: Validation and investigation. SC: Investigation. JW: Writing-review and editing, and funding acquisition. All authors contributed to the article and approved the submitted version.

## Funding

This work was supported from the following funds: the National Key R&D Program of China (Project No. 2017YFD0502200), the program for the Beijing Dairy Industry Innovation Team and the National Natural Science Foundation of China (Project No. 31873034).

## Conflict of Interest

The authors declare that the research was conducted in the absence of any commercial or financial relationships that could be construed as a potential conflict of interest.

## Publisher’s Note

All claims expressed in this article are solely those of the authors and do not necessarily represent those of their affiliated organizations, or those of the publisher, the editors and the reviewers. Any product that may be evaluated in this article, or claim that may be made by its manufacturer, is not guaranteed or endorsed by the publisher.
