# Supplementary material for: Lactobacillus rhamnosus GR-1 Prevents Escherichia coli-Induced Apoptosis Through PINK1/Parkin-Mediated Mitophagy in Bovine Mastitis
Source: Front Immunol. 2021 Sep 14;12:715098. doi: 10.3389/fimmu.2021.715098 (PMC8476910; doi:10.3389/fimmu.2021.715098)
Supplement: Supplementary file 1 [file Table_1.pdf]

Table S1 Primers used in this study.

Gene	Primer	Sequence 5′→3′
β-actin	Forward	GCTCTTTTCCAGCCTTCCTT
	Reverse	GATGTCAACGTCACACTT
IL-1β	Forward	ATGAAAGACGGCACACCCAC
	Reverse	GCTTGTGCTCTGCTTGTGAG
TNF-α	Forward	ACGGCATGGATCTCAAAGAC
	Reverse	AGATAGCAAATCGGCTGACG
